# Awareness of middle ear pathologies in South Africa: Towards a primordial preventive audiology

**DOI:** 10.4102/sajcd.v71i1.1026

**Published:** 2024-09-30

**Authors:** Ben Sebothoma, Naledi Baloyi

**Affiliations:** 1 Department of Speech Pathology and Audiology, Faculty of Humanities, University of the Witwatersrand, Johannesburg, South Africa

**Keywords:** awareness, middle ear pathologies, risk factors, primordial prevention, South Africa

## Abstract

**Background:**

Public awareness of auditory pathologies, has been explored in the literature. However, there is limited evidence regarding public awareness of middle ear pathologies and their risk factors in South African communities.

**Objectives:**

The aim of this study was to describe public awareness regarding middle ear pathologies and their associated risk factors in the community of Giyani, Limpopo province, South Africa.

**Method:**

A quantitative cross-sectional survey design was conducted among 94 adults aged 18 years and older living in Giyani, Limpopo province. A questionnaire was used to collect data. Descriptive statistics was used to summarise the data, while a Chi-squared test was used to determine if there is any association between awareness and independent variables with categorised outcomes.

**Results:**

Adults aged between 18 and 65 years participated in this study. Most participants were aware of aural itchiness (71.3%) and pain (35%) as symptoms related to middle ear pathologies. The use of cotton buds (51.1%) and other illnesses (35.5%) were primarily reported as risk factors for middle ear pathologies. There were varied responses with regard to awareness of which professionals manage middle ear pathologies, indicating a general lack of awareness. No statistically significant association was found between the dependent and independent variables (*p* > 0.005).

**Conclusion:**

The study indicated a general lack of public awareness regarding middle ear pathologies and their associated risk factors among Giyani community.

**Contribution:**

The study raises implications for public awareness campaign that educates communities about middle ear pathologies, risk factors and social determinants of health associated with these pathologies.

## Introduction

Public awareness of auditory pathologies is a topic of scholarly importance, especially in the context of preventive audiology. Such awareness helps communities to recognise the condition of interest, including their symptoms and associated risk factors. Resultantly, if communities are aware of health-related conditions, this can significantly improve early identification and interventions (Khoza-Shangase, 2022), especially in the context of low- and middle-income countries (LMICs) where resources to manage these conditions are lacking (Fagan & Jacobs, 2009; Pillay et al., 2020). While several studies have explored public awareness of various auditory pathologies such as hearing loss (Alanazi & Al Fraih 2021; Alshehri et al., 2019; Joubert et al., 2017), there is limited evidence on public awareness of middle ear pathologies. The dearth of evidence regarding public awareness of middle ear pathologies is concerning given that the World Health Organization (WHO) has reported that approximately 700 million people live with middle ear pathologies (WHO, 2021).

In LMICs, the prevalence of middle ear pathologies is also reported to be significantly higher, accounting for 30% of the total global prevalence (WHO, 2021). In South Africa, the prevalence of middle ear pathologies has also been reported to be high (Ramma & Sebothoma, 2016; Sebothoma & Khoza-Shangase, 2022). The persistence of middle ear pathologies can result in a number of other problems such as hearing loss. Mulwafu et al. (2016) reported that 36% of the hearing loss in LMICs results from untreated middle ear pathologies. The WHO has reported that approximately half of individuals with untreated acute middle ear pathologies will develop chronic suppurative otitis media (CSOM) (WHO, 2021), which often requires specialised services such as those offered by ear, nose and throat (ENT) specialists. Choi and Park (2015) also noted that untreated middle ear pathologies can cause facial paralysis and brain problems.

While subtle or less severe form of middle ear pathologies can be treated successfully at the primary level care, services to effectively treat chronic middle ear pathologies and middle ear sequalae are extremely limited (Fagan & Jacobs, 2009). Indeed, the scarcity of these specialised services poses significant challenges for people living in rural and township areas to access them. This can result in a significant delay in receiving hearing healthcare service, affecting the identification and intervention of middle ear pathologies. In addition, limited access to specialised services may affect help-seeking behaviour (Mtimkulu & Khoza-Shangase, 2023), resulting in community members resorting to readily available methods that may be hazardous for hearing health (Joubert et al., 2017).

Public awareness can play a crucial role in preventive audiology, particularly in the early identification and intervention of middle ear pathologies. The WHO (2018) emphasised the importance of raising public awareness in order to facilitate early identification of hearing health conditions, such as middle ear pathologies. Creating public awareness may influence policymakers to implement strategies at primary and primordial level of care (Khoza-Shangase, 2022). At the primary level of care, public awareness may focus on ensuring that communities have the necessary information for simple preventive strategies, promoting health and seeking hearing health for early identification and intervention (Chadha, 2013). Primordial level of care focuses on reducing risks at the population level (Khoza-Shangase, 2022). While the latter may be difficult to achieve, especially in resource-constrained environments with high burden of diseases (Moroe, 2022), public awareness may influence national policy to address the issues that cause middle ear pathologies to persist in the community.

Given the importance of public awareness data about audiological services and auditory pathologies, this study intends to extend the knowledge by investigating public awareness of middle ear pathologies, their associated symptoms and risk factors in a community of Limpopo province, South Africa. The study hypothesises that the community of Giyani in Limpopo province has a higher awareness of middle ear pathologies.

### Aim and objectives

The aim of this study was to describe public awareness of middle ear pathologies in the community of Giyani, Limpopo province, South Africa. The objectives were to: (1) describe the awareness of symptoms associated with middle ear pathologies in community of Giyani, Limpopo province; (2) describe the awareness of risk factors associated with middle ear pathologies in community of Giyani, Limpopo province; (3) Describe awareness of healthcare professionals responsible for middle ear assessment and management and (4) determine the association between symptoms and/or risk factors and other demographic factors.

## Research methods and design

### Study design

A quantitative cross-sectional research survey design was used to investigate public awareness of middle ear pathologies, associated symptoms, risk factors and information needs of the communities. Quantitative research designs are useful in collecting numerical data (Watson, 2015) and using statistical methods to examine the relationship between variables such as demographic characteristics of the participants and awareness of middle ear pathologies (Irwin et al., 2014).

### Setting

This study was conducted in Limpopo province, one of the nine provinces in South Africa, which has approximately 6 million people (Statistics South Africa, 2022). In this province, audiological services and audiologists are limited (Pillay et al., 2020), with only 4% of the health sector in Limpopo providing audiological services (Maluleke, 2021; unpublished). There are many socioeconomic challenges in the province, including high levels of unemployment and poverty, low literacy and educational levels (Stats SA, 2022; Tambe et al., 2023). As a result, the community of Giyani was selected as the study setting because it represents a typical South African rural area with myriads of challenges and scarcity of hearing health services.

### Study population and sampling strategy

A probability systematic random sampling strategy was employed to recruit participants (Polit & Beck, 2010). In a systematic random sampling strategy, participants are selected based on a system of intervals in a numbered population (Bhardwaj, 2019). For the purpose of this study, simple systematic random sampling strategy was used to select every third home in Giyani community. This type of sampling strategy has previously been used in audiology survey research (Joubert et al., 2017; Kisten et al., 2022). This sampling strategy has several benefits, including moderate costs, high internal and external validity, and ease of verification (Acharya, et al., 2013). Within homes, the researcher asked at least one individual who met the inclusion criteria to participate. Inclusion criteria involved adults aged 18 years and above who were part of the visited homes. Participants were excluded if they were ill on the day of the survey. Of the approximately 26 000 homes in the study population, and using a confidence level of 95%, a power of 80% and a margin of error of 5%, the estimated sample size was approximately 380. However, a total of 94 adults in 94 homes were surveyed.

### Data collection

Data collection was conducted using a self-administered questionnaire. This questionnaire consisted of 5 sections and a total of 15 items (questions). The sections included the demographic information of the participants, awareness of the symptoms related to middle ear pathologies, awareness of professionals responsible for assessment and management of middle ear pathologies, and awareness of risk factors for middle ear pathologies. The items (questions) on the questionnaire were primarily closed-ended in nature, with an option of ‘other’ for those who wanted to provide more information. This questionnaire was developed based on previous research (Alanzi & Fraih, 2021; Berardino et al., 2013; Joubert et al., 2017), but were modified to suit this specific research. The questionnaire was piloted with five adults using the same procedure as the main study. This process allowed the research to determine the feasibility of the questionnaire, including the language used. Some community members and leaders were also asked to review the questionnaire and commented on the linguistic and cultural appropriateness of the questions. The questionnaire was then administered to participants who volunteered to participate and signed the consent form. For participants who were literate, the researcher (second author) provided the questionnaire for self-completion. For participants who could not read, the researcher read the statements or questions and options aloud, allowing them to select the most appropriate options. However, for participants who could not write, the researcher administered the questionnaire reading the statements and allowing the participants to choose the most appropriate answer. The researcher administered the questionnaire by asking questions on the questionnaires. Data collection took approximately 4 weeks to complete.

### Data analysis

Raw data were imported into an Excel spreadsheet (Microsoft Office 360) and then converted to STATA software version 15.2 for analysis. Data cleaning was performed by dropping any missing values and unnecessary variables. Descriptive analysis was then conducted to summarise all the data (Pagan et al., 2022). Frequencies and percentages for all categorical data were used. Inferential statistics was used to determine if there were any associations between the dependent variables (e.g. awareness of middle ear pathologies) and independent variables such as demographic information. Because the outcomes of the dependent and independent variables were categorised, a Chi-squared test was used to establish if this association exists, under the research hypothesis that there is association between awareness of middle ear pathologies and certain independent variables, with significance level set at *P* < 0.005.

### Ethical considerations

The community targeted comprised approximately 26 000 homes. Before this study was conducted, ethics clearance was obtained from the Human Research Ethics Committee (HREC) (reference number STA_2023_29) of the University of the Witwatersrand, Johannesburg, South Africa. Permission to conduct the study was sought from the Giyani municipality. Potential participants were informed about the study via word of mouth by community leaders and other community members. Flyers were also distributed at strategic points where it was visible to community members. The municipality was provided with a detailed information letter that provided an overview of the study, including the nature and purpose of the study, eligibility criteria of adults aged 18 years and above, and the possible duration of the study. Community leaders were also informed about the details of the study. Because homes were randomised, community members were not asked to indicate their participation before the study began. The researcher entered a home using a randomised process set for this study and asked if they would like to take part in the study. Consent to participate was obtained after participants in the home understood the details of the study and agreed to participate. Obtaining informed consent was not an issue, as the researcher spoke the language of the community (O’Sullivan et al., 2021).

## Results

**FIGURE 1:**
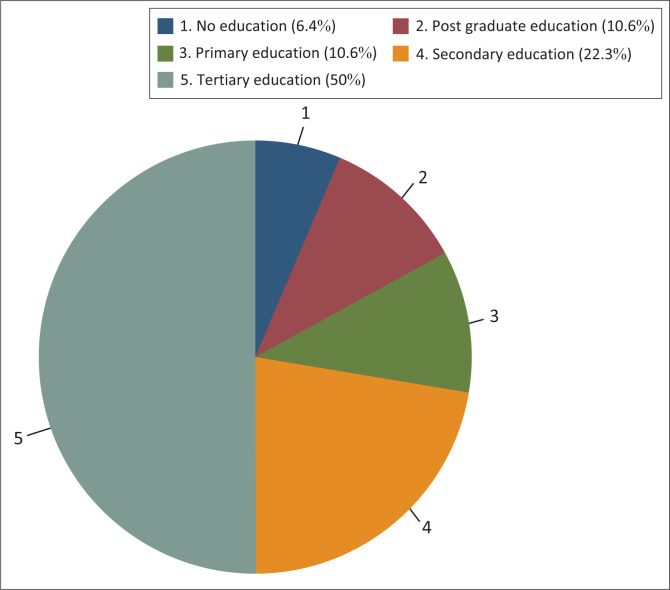
The education level distribution of the participants.

**FIGURE 2:**
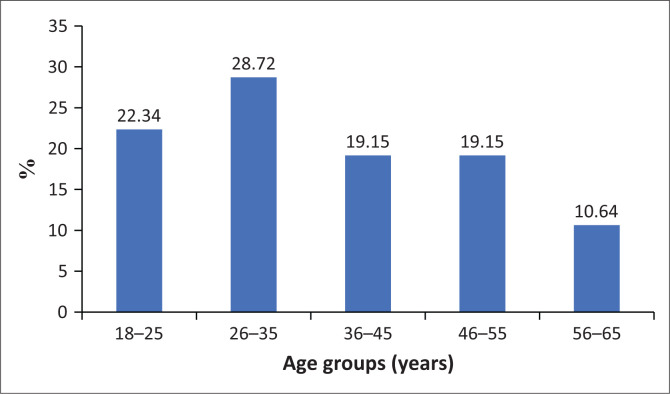
The age group distribution of the participants.

Ninety-four adults (*n* = 94) participated in the study. A little over half (57%) of the participants were males, while 43% were females, with no statistically significant difference between the two groups (*p* > 0.005). Majority of the participants (91.5%) spoke Xitsonga, which is the predominant language of the community. Half of the participants (50%) had attained some tertiary education, while the rest had variety of education levels (Figure 1). Figure 2 shows the age group distribution of the participants, with most participants aged ranging between 26 and 35 years.

### Objective 1: Describe the awareness of the symptoms of middle ear pathologies among the community

Table 1 shows the responses of the participants with regard to the awareness of symptoms associated with middle ear pathologies. About 71.3% indicated that itching, pain in the ear (35.1%), discharge (pus) (4.3%) and feeling of fullness (13.8%) were symptoms related to middle ear pathologies. When participants were asked if they were aware of other individuals with symptoms related to middle ear pathologies, 35.1% indicated they knew some with itching ear, painful ears (32.3%), discharge ears (13.8%) and only a few were aware of people experiencing a feeling of fullness (8.5%) in the ear. Some participants (21.2%) indicated that those who had presented with symptoms related to middle ear pathologies were friends, family members (35.1%) or community members (12.8%).

**TABLE 1:** Awareness of middle ear pathologies’ symptoms.

Awareness	Variable	Categories	Frequency	%
Awareness of symptoms	Itchiness	No	27	28.72
		Yes	67	71.28
	Pain	No	61	64.89
		Yes	33	35.11
	Discharge	No	90	95.74
		Yes	4	4.26
	Feeling of fullness	No	81	86.17
		Yes	13	13.83
Knowledge of anyone with MEP Symptoms	Itchiness	No	61	64.89
		Yes	33	35.11
	Pain	No	63	67.74
		Yes	30	32.26
	Discharge	No	81	86.17
		Yes	13	13.83
	Feeling of fullness	No	86	91.49
		Yes	8	8.51
Who do you know?	Friend	No	74	78.72
		Yes	20	21.28
	Family member	No	61	64.89
		Yes	33	35.11
	Community	No	82	87.23
		Yes	12	12.77
	Other	No	92	97.87
		Yes	2	2.13

MEP, middle ear pathologies.

### Objective 2: Descriptions of awareness regarding risk factors related to middle ear pathologies

Table 2 shows the risk factors associated with middle ear pathologies. About 3.2% of the participants were aware that second-hand smoking is a risk factor for middle ear pathologies, while 6.4% and 18.1% were aware of overcrowding and trauma as risk factors, respectively. Majority of the participants (51.1%) were aware that the use of cotton buds can contribute to the development of middle ear pathologies. Some participants (35.2%) indicated that some illnesses may be risk factors for middle ear pathologies.

**TABLE 2:** Risk factors for middle ear pathologies.

Variable	Categories	*n*	%
Second-hand smoking	No	91	96.81
	Yes	3	3.19
Overcrowding	No	88	93.62
	Yes	6	6.38
Trauma	No	77	81.91
	Yes	17	18.09
Cotton buds	No	46	48.94
	Yes	48	51.06
Other illnesses	No	61	64.89
	Yes	33	35.11

### Objective 3: Description of awareness of professionals responsible for middle ear assessment and management

**FIGURE 3:**
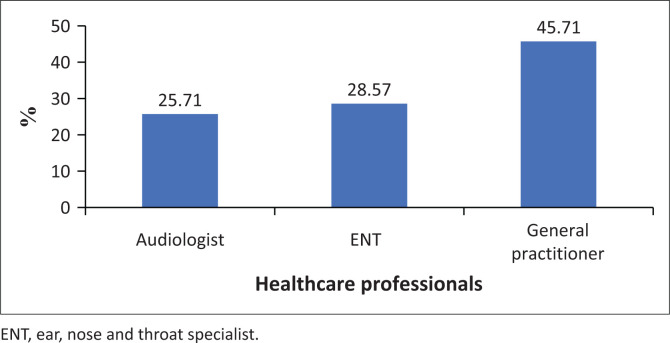
Distribution of knowledge of professionals who assess and manage middle ear pathologies.

Figure 3 shows the distribution of the professionals who assess and manage middle ear pathologies. Almost half (45.7%) indicated that general medical practitioners (GPs) assess and manage middle ear pathologies. About 28.6% and 25.7% indicated that they were aware that otorhinolaryngologists (ENTs) and audiologists, respectively, assess and manage middle ear pathologies.

### Objective 4: Association between demographic characteristics and awareness of middle ear pathologies

Table 3 and Table 4 show a distribution of the association measurements between the demographic characteristics of gender and level of education, and awareness of middle ear pathologies (*p* > 0.005). Chi-squared test indicated that there was no association between any of the variables measured.

**TABLE 3:** Association between gender and symptoms of middle ear pathologies.

Variable	Categories	Female		Male		*p*
		*n*	%	*n*	%	
Itchiness	No	19	70.37	8	29.63	0.567
	Yes	43	64.18	24	35.82	-
Pain	No	41	67.21	20	32.79	0.727
	Yes	21	63.64	12	36.36	-
Discharge	No	59	65.56	31	34.44	0.696
	Yes	3	75.00	1	25.00	-
Feeling of fullness	No	54	66.67	27	33.33	0.717
	Yes	8	61.54	5	38.46	-

**TABLE 4:** Association between level of education and symptoms of middle ear pathologies.

Variable	Categories	No education		Post-graduate		Primary		Secondary		Tertiary		*p*
		*n*	%	*n*	%	*n*	%	*n*	%	*n*	%	
Itchiness	No	2	7.41	2	7.41	3	11.11	5	18.52	15	55.56	0.92
	Yes	4	5.97	8	11.94	7	10.45	16	23.88	32	47.76	1.00
Pain	No	2	3.28	5	8.20	6	9.84	15	24.59	33	54.10	0.32
	Yes	4	12.12	5	15.15	4	12.12	6	18.18	14	42.42	2.00
Discharge	No	6	6.67	9	10.00	8	8.89	21	23.33	46	51.11	0.07
	Yes	0	-	1	0.25	2	50.00	0	-	1	25.00	1.00
Feeling of fullness	No	4	4.94	8	9.88	10	12.35	19	23.46	40	49.38	0.37
	Yes	2	15.38	2	15.38	0	-	2	15.38	7	53.85	8.00

## Discussion

The purpose of this study was to investigate and describe public awareness of middle ear pathologies, symptoms associated with middle ear pathologies and risk factors among community members residing in Giyani, Limpopo province. Although previous studies have described public awareness of other auditory pathologies such as hearing loss and demonstrated that there is a general lack of awareness of auditory pathologies (Alnuman & Ghnimat, 2019; Joubert et al., 2017; Mahomed & Panday, 2024), this is one of the first studies that described awareness of middle ear pathologies and related risk factors.

Community members were generally aware of symptoms associated with middle ear pathologies. Participants were mostly aware of itchiness and pain as the main symptoms related to middle ear pathologies. Very few participants reported feeling of fullness and discharge as the symptoms related to middle ear pathologies. These findings were not surprising given that itchiness and pain seem to be some of the common symptoms for middle ear pathologies, indicating an onset of the pathology (Martin & Clarke, 2019). In a study conducted in Nigeria, Musa et al. (2015) found that just over 50% of the participants presented with ear pain and itchiness as a major complaint. Similar studies have also indicated that ear itchiness is a common symptom among adult population (Olajide et al., 2015; Vallur et al., 2021).

Few participants were aware of other people presenting with or complaining of symptoms related to middle ear pathologies. About 32.3% indicated that they were aware of other people who presented with pain, while 13.8% were aware of people presenting with discharging ears. These findings were not surprising given that people who present with pain and/or discharging ears can easily be seen (Gaddey et al., 2019), as they constantly scratch their ears or/and complain about the symptom, especially if they are close friends and family members. Furthermore, people who experience middle ear symptoms such as pain or ear discharge may report these symptoms to family members or friends.

Awareness of risk factors associated with middle ear pathologies was varied, but generally poor among the community members. Participants in this study were mostly aware of the use of cotton buds (51.1%) and ‘other illnesses’ (35.15%) as risk factors. However, participants did not specify which illnesses contribute to the development of middle ear pathologies because this information was also not elicited from the participants, which requires further exploration. Several studies have indicated that medical conditions such as human immunodeficiency virus (HIV), which are epidemic in LMICs (UNAIDS, 2022), are associated with middle ear pathologies and its related symptoms (Khoza-Shangase & Anastasiou, 2020; Sebothoma & Khoza-Shangase, 2020; Van der Westhuizen et al., 2013).

This study also showed that community members in Giyani are not aware of health professionals who assess and manage middle ear pathologies. Less than 30% of the participants indicated that audiologists assess middle ear, while approximately 46% of the participants reported that general practitioners assess and manage middle ear pathologies. These findings are consistent with a study by Joubert et al. (2017) who indicated that majority of the communities are not aware of audiologists. Not knowing or being aware of hearing health professionals can lead to delayed identification and intervention of middle ear pathologies (Sebothoma & Khoza-Shangase, 2022), resulting in permanent auditory damage with the potential to significantly affect the quality of life (Swain, 2022) and incurring high costs for assessment and management (McDaid et al., 2021; Thai et al., 2022).

The findings of this study indicate that community members, particularly in underserved areas, are not aware of the symptoms associated with middle ear pathologies and the risk factors related to middle ear. Information deficit regarding middle ear pathologies was also evident among the participants. Lack of public awareness has implications for early identification and intervention of middle ear pathologies (Sebothoma & Khoza-Shangase, 2022). Literature has indicated that when communities are not aware of and have information deficits about auditory pathologies and treatment options, they may resort to hazardous methods (Joubert et al., 2017). Therefore, providing tailor-made awareness campaigns may help communities to seek hearing health and prevent the sequalae of untreated middle ear pathologies.

The present findings further provide evidence for the need of effective preventive audiology strategies, especially in LMICs where resources are generally scarce. While the WHO suggests that prevention of middle ear pathologies must focus on early identification to prevent the development of chronic otitis media (WHO, 2021), current authors argue that effective management of middle ear pathologies should involve a primordial preventive strategy. These strategies must aim at averting risk factors for middle ear pathologies. Middle ear pathologies have been associated with risk factors related to social determinants of health (Delacy et al., 2020). Therefore, in line with the sustainable development goal, whose intentions are to address social determinants of health (Stats SA, 2023), public awareness campaigns may play a crucial role in educating the society, addressing some of these risk factors (Pazhayapisharath & Maruthy, 2023). In addition, government may also need to implement public health policies to reduce risk factors associated with middle ear pathologies.

### Limitations

Despite the important evidence presented in this current study, there are some limitations that require findings to be interpreted with cautions. One important limitation for this study is small sample size, which has the potential to affect generalisation of the findings. This study was also conducted in one community in a single province, which has slightly different demographics and other socioeconomic factors than other provinces within the country and other countries. There were further methodological limitations. Participants were not asked to provide detailed information about their awareness of middle ear pathologies because of the use of a quantitative research. Instead, participants were given mostly closed-ended questions. A qualitative study could be used in future research to allow participants to provide detailed information about their awareness of middle ear pathologies. While the sampling strategy used in this study is relatively strong, those who were not found in their homes may have been excluded from participation. Future studies should focus on addressing the limitations of this study.

## Conclusion

Middle ear pathologies remain high and continue to rise especially in LMICs (WHO, 2018). This current study indicated that despite the high prevalence of middle ear pathologies and the multiplicity of risk factors, many community members surveyed were generally not aware of the symptoms and risk factors of middle ear pathologies and had varied responses regarding the professionals responsible for dealing with these pathologies. While studies have indicated the importance of awareness campaigns (Joubert et al., 2017; Mukara et al., 2017), current authors argue for public awareness campaigns that focus on primordial care such as addressing social determinants of health that are associated with middle ear pathologies (DeLacy et al., 2020).
